# Building bridges: evaluating policymakers' research capacities, engagement, and utilization in health policymaking within the Kuwaiti context: a cross-sectional study

**DOI:** 10.1186/s12961-024-01177-9

**Published:** 2024-07-15

**Authors:** Abdulaziz Alhenaidi, Asmaa Al-Haqan, Heba Alfarhan, Limya Alaradi, Mohamed Elsherif, Hisham Kelendar

**Affiliations:** 1 Ministry of Health, Kuwait City, Kuwait; 2 General Practice and Primary Care, School of Health and Wellbeing, University of Glasgow, Glasgow, Scotland; 3 Pharmacy Practice Department, College of Pharmacy, Kuwait University, Kuwait City, Kuwait; 4 Division of Gastroenterology, Department of Internal Medicine, Thunayan Al Ghanim Gastroenterology Center, Al Amiri Hospital, Ministry of Health, Kuwait City, Kuwait

**Keywords:** Kuwait, Health policy, Evidence-based practice

## Abstract

**Background:**

Health policymaking is a critical aspect of governmental decision-making that shapes the well-being of populations. In the Middle East and North Africa, particularly in Kuwait, limited attention has been given to exploring the research capacities, engagement, and utilization among health policymakers. This study aims to bridge this gap by investigating how Kuwaiti health policymakers incorporate evidence-based research into the formulation of health-related policies.

**Methods:**

This cross-sectional study targeted health policymakers in leadership positions within the Kuwait Ministry of Health (MOH). Using the Seeking, Engaging with and Evaluating Research (SEER) questionnaire, participants' capacities, engagement, and use of research were assessed. The targeted sample was all health policymakers in leadership positions, starting from the head of departments and above. The questionnaire comprises four domains, 14 sections, and 50 questions and utilizes Likert and binary scales, with aggregate scores predicting engagement actions and research use. The data were collected between March and July 2023. All the statistical analyses were performed using SPSS v27, and the numerical and categorical variables were analyzed using appropriate statistical tests, including t-tests, ANOVA, and Pearson's correlation.

**Results:**

Out of 205 policymakers, 88 participated (42.9% response rate): predominantly male (51.1%) and married (78.4%). The mean age was 49.84 ± 7.28 years, with a mean MOH tenure of 24.39 ± 6.80 years. Participants demonstrated high value for research (mean score 4.29 ± 0.55) and expressed confidence in the research utilization. Organizational emphasis on research use exhibited nuanced perceptions, identifying areas where MOH support may be lacking. Access to research resources and processes for policy development guidance were highlighted as challenges.

**Conclusions:**

This study provides crucial insights into the research capacities and engagement of Kuwaiti health policymakers. It emphasizes the need for targeted interventions to align individual perceptions with organizational expectations, address confidence disparities, and enhance collaborative efforts. Organizational investments are crucial for fostering a dynamic research ecosystem to improve evidence-based policy development in Kuwait's healthcare landscape.

**Supplementary Information:**

The online version contains supplementary material available at 10.1186/s12961-024-01177-9.

## Background

Decision-makers at the national and governmental levels influence our lives through the policies they implement. A crucial aspect of these policies is the formulation and implementation of health policies, which are vital for developing health systems that more effectively address the health needs of their populations [1]. It is widely recognized that research can significantly contribute to the development of health policies. Utilizing robust evidence to guide public health policy is likely to achieve greater and more equitable health benefits for the population [2].

Even though evidence-based research is an important driver of decision-making, community characteristics, needs, and preferences should also be considered [3]. Therefore, other data sources, such as needs assessments, population characteristics, community resources and values, ideas and interests, and professional and practical experience, have proven to be essential in the decision-making process, all in a broader environmental and organizational context [4, 5]. One of these policymaking approaches that uses research and other data sources is called evidence-informed policymaking (EIP), and the global dedication to using it has grown significantly in the past twenty years. This approach is believed to maximize equitable health outcomes for the population, enhance the cost-effectiveness of health services and policies, and improve transparency and accountability in decision-making processes [6]. By using EIP, policymakers can use research evidence to design interventions that best fit their communities.

Several factors must be considered to ensure the effective use of research in health policymaking. Health policy and systems research focuses on understanding and improving how societies are organized to achieve collective health objectives. Key characteristics include its diverse participants, practical nature, engagement with structural changes, commitment to social justice principles, and multidisciplinary perspective. Therefore, developing the health policy and systems research field emphasizes enhancing connections among individuals and organizations, ensuring collaboration across different communities of practice, and consolidating and expanding shared ideas and experiences [1].

Moreover, for research findings to be effectively transformed into meaningful policies, they must be relevant, timely, accessible, and clear. Without these factors, even robust research evidence will not lead to well-informed policies [7, 8]. Policymakers must have confidence in research and the capacity to understand and apply it, as well as appreciate its value [4]. This highlights the importance of policymakers' qualities and capacities in evidence-informed policymaking. Numerous studies have shown that these personal qualities and research capacities are crucial for the effective use of research evidence in public health policymaking [3, 4].

Other factors that also play a role are related to the organizational culture within the decision-making organization and whether it harbors a positive research culture and a supportive environment that enables and supports research to generate new knowledge and translate research into practice [9, 10]. Therefore, if one wishes to determine the extent to which research is implemented successfully in the decision-making process, then a good start would be to study the research capacity of the decision-makers and the support they get from their organization to translate research into policies. In their research on policymakers, Newman et al. (2012) and Brennan et al. (2017) defined policymakers’ capacity as a widely used term conceived of a multilevel concept (individual, organizational, enabling environment) encompassing four elements: tools, skills, staff, and infrastructure (and roles in it), increasingly used to build competencies to implement evidence-based practice [11, 12]. As previously defined, research capacity is still not a widely explored concept in the literature, and it is still challenging to measure it with no concrete agreement on its definition [4].

A 2018 review of the Kuwait Health System concluded that strategic policymaking decisions in Kuwait are not evidence-based and may be somewhat politically motivated. In addition, health policy decisions are developed without sufficient consultation with stakeholders and their execution is not well planned. Although electronic healthcare records exist within primary healthcare centers, it is unclear how the MOH uses collected data to develop evidence-based policies. This lack of evidence-based policymaking in the healthcare sector has hampered the country’s ability to adapt to new challenges caused by rising rates of non-communicable diseases, falling revenues and technological changes [13]. Consequently, the system has not been able to adapt to the population's changing needs. In addition to the latter, key governmental decision-makers (and/or their advisers) do not always have experience in health policy, health management, health economics and/or public health and may have limited research capacities, which is a limitation when developing appropriate policies [13]. Clearly, there is an area of improvement in the use of research within policymaking in Kuwait. However, the first step in improving this approach is to assess the use of evidence-based research among Kuwaiti health policymakers. This will constitute a baseline upon which any future action should be built.

Particularly in the Middle East, there is little research on health policymakers’ research capacities, making this paper a vital milestone in studying how health policies are shaped in the region. This paper aims to explore how Kuwaiti health policymakers make use of evidence-based research when they are developing health-related policies. We aim to examine their capacities, engagement, and research use in health policymaking.

## Methods

### Study design, settings, and targeted population

This cross-sectional study aimed to investigate the research capacity, engagement, and use of research among health policymakers in Kuwait. The targeted sample was all 205 health policymakers in leadership positions, starting from the head of departments and above, which include (e.g., minister, undersecretary, assistant undersecretary, directors of central directorates, heads of departments and supervisors in central directorates, heads of clinical specialty councils), and directors of different catchment areas, Kuwait Institute of Medical Specializations, and the Legal Responsibility Authority. To avoid selection bias, we targeted all health policymakers. The exclusion criterion was working in a nonclinical area (financial or administrative sectors) or a researcher working within a university or other research organization during the data collection. The existing organizational structure of the Kuwait Ministry of Health was used to identify the eligible participants who were contacted and received a copy of the questionnaire (a link to the electronic version or hardcopy according to their preference) with two-weeks reminders. The data were collected between the end of Mars and the end of July 2023.

### Measurement

Data was collected using the SEER questionnaire [12]. The questionnaire assesses individual policymakers’ (1) capacity to use research/evidence (predisposing factors), (2) research/evidence engagement actions and (3) actual research use (instrumental, conceptual, tactical, imposed) [12]. The questionnaire was validated and used in Australia [12] and Denmark [4]. The SEER questionnaire was developed as a part of the Supporting Policy In health with Research: an Intervention Trial (SPIRIT) Action Framework [12], which was created to guide informed decisions in selecting and testing interventions aimed at increasing research use in policy [14]. SEER is one of three tools in the SPIRIT Action Framework [14].

The tool consists of four domains, 14 sections, and 50 questions. The first domain assesses the capacity of policymakers to use research and consists of 4 sections; the scoring of the first two sections is a five-point scale ranging from (not at all valuable—score = 1) to (very valuable—score = 5), the third section is a five-point scale ranging from (never—score = 1) to (always—score = 5). The last section is a four-point scale scored as (no—score = 1), (yes, but limited—score = 2), (yes well developed—score = 3), or (I don’t know—recorded as ‘no’). The second domain examines research engagement actions and has five sections; four sections have binary scales, and the last section is a four-point scale ranging from (not at all—score = 1) to (more than twice—score = 4). The third domain examines the extent of research use and has one section scored on a six-point scale ranging from (none—score = 1) to (extensive—score = 6). The final domain assesses the type of research used and has four sections with binary scales [12]. In addition, the survey included a section consisting of eight questions to collect basic sociodemographic data. The SEER instrument was minimally adapted for the Kuwaiti context; one change was made to the section of (accessed synthesized research) in the third question (commission a review of research to summarize and evaluate the results of available studies?) the word (commission) was replaced by (headed or delegated). In addition, an example was added from Kuwait to the question under the section (interacted with researchers) in the third question (collaborated on a competitive research grant application).

### Data analysis

Statistical analysis was done using SPSS v27 (IBM Co., Armonk, NY, USA). Numerical data are presented as mean with standard deviation (SD) and were analyzed using independent samples t-test or one-way ANOVA as appropriate after confirmation of the normality of distribution using the Shapiro–Wilk test. Categorical variables were presented as frequency and percentage (%). Pearson’s correlation was used to evaluate the associations between age, duration of working in MOH, and different variables. There were no missing data. We chose to report the mean score as there is much variation in the total score of each domain, and a *p*-value of < 0.05 was considered to indicate statistical significance.

## Results

Out of 205 policymakers, 88 participants were included in this study (response rate 42.9%), of whom almost half were males, 45 (51.1%), and 69 (78.4%) were married. Forty participants (45.5%) received their highest education from Kuwait, and 38 (43.2%) received their highest education from Western and other countries. The mean duration of working in MOH was 24.39 ± 6.80 years. The participants' demographics are available in the additional file.

Regarding the value individuals place on using research, the mean score was 4.29 ± 0.55. Almost None of the participants categorized any statements as not valuable at all, and a few as not valuable. The statement "Meet organizational requirements to use research" was labelled neutral by many participants compared to other statements; however, the statement was considered valuable by the highest number of participants. Approximately half of the participants considered the statement "Monitor implementation or evaluate the impact of a policy or program" very valuable (Additional file). In relation to the confidence the participants had in their knowledge and skills, the mean score was 3.78 ± 0.70. The highest statement that received "not confident" or "neutral" labels was the "Evaluate the quality of research" statement. On the other hand, the participants were confident in "Apply research to policy or program development", "Commission research to support policy or program development", and "Partner with researchers to generate research". More than a quarter of the participants were very confident in "Find research to inform policy or program development"; details are available in the additional file.

In terms of the value the MOH places on using research, the mean score was 3.30 ± 0.88. Eight percent of the participants labelled the statement "Interaction or collaboration with researchers or research organizations is encouraged" "never", and more than a quarter of the sample thought that "Generation of new research to inform policy or program development is encouraged" was rarely applied to the MOH. Approximately one-third of the sample recognized that sometimes "Leaders believe it is important to use research in policy or program development" and "It is expected that policies/programs will be evaluated". In addition, (30.7%) thought that in the MOH, often, "It is expected that research will be used in policy or program development", and (17%) recognized that leaders always believe it is important to use research in policy or program development (Additional file).

Regarding the tools and systems organization that support research engagement actions and use, the mean score was 1.67 ± 0.54. More than 50% of the sample thought there was no access to the needed research resources. In addition, 50 participants recognized that there are limited “Processes for policy or program development that provide guidance on how research should be used”. Only 13 participants thought that there are well-developed “Processes for policy or program development that provide guidance on how research should be used”, “Access to training in using research in policy or program development”, and “Relationships, or established methods for engaging, with research organizations” (Additional file).

Concerning access to synthesized research, 62 (70.5%) participants searched for "Reviews of research summarizing and evaluating the results of multiple studies", and 53 (60.2%) "Headed or delegated a team to summarize and evaluate the results of available studies". The respondents' answers to questions related to access to primary research showed that 58 (65.9%) participants searched for research papers reporting the results of single studies and that 47 (53.4%) searched for research on government websites. Regarding research appraisal experience, 56 (63.6%), 58 (65.9%), and 60 (68.2%) mentioned that they participated in appraisal in "The appropriateness of methods used to answer the question", "The likelihood that the methods used meant that the results were reliable (unbiased)", and "Generalizability of the findings to your context, based on similarity of the included population, health system or other factors", respectively (Additional file).

Regarding the participants' research generation, 61 (69.3%) had undertaken or participated in an internally conducted research project or analysis of data, 59 (67%) commissioned or partnered with researchers to conduct a research project or analysis of data, and 58 (65.9%) planned or undertaken an evaluation of the program or policy. As of the interaction with other researchers, the mean score was 2.07 ± 0.92, in which 61 participants (69.3%) never collaborated on a competitive research grant application. Additionally, approximately 24% of the participants contributed to the analysis and/or writing up of research results or to other aspects of a research publication only once, 15 (17%) collaborated with researchers to develop or implement a research project twice, and 31 (35.2%) attended forums to hear about research findings more than twice (Additional file).

Regarding the extent of use of the research, the mean score was calculated and was 2.54 ± 1.29. Sixteen participants (18.2%) thought it was minimally used in policy or program development, 17 (19.3%) recognized it limited usage in policy or program evaluation, and 29 (33%) mentioned that it was used to a moderate extent in agenda setting/scoping (Additional file). Regarding the type of research used by the respondents, the most common type was conceptual research (56; 63.6%), and the least common type was imposed research by the organization (Additional file).

There was no statistically significant association between participants' demographics and the mean value individuals placed on using research or the participants' confidence in their knowledge and skills. There was no correlation between age or duration of working in MOH, the mean value individuals place on using research, or their confidence in their knowledge and skills (Additional file). However, there was a statistically significant association between gender and the mean value that organization places on using research, as the mean value that organization places on using research for males (3.10 ± 0.91) was lower than the mean value that organization places on using research for females (3.50 ± 0.80), *P* = 0.034. In addition, there was a positive moderate correlation between age and the mean value of organization places on research (Pearson correlation coefficient = 0.297, *P* value = 0.005). Additionally, a positive moderate correlation was observed between the duration of working in MOH and the mean value the organization places on using research (Pearson correlation coefficient = 0.353, *P* value = 0.001) (Additional file).

There was no statistically significant association between participants' demographics or mean interaction with researchers' mean extent of research use. Similarly, no correlation existed between age or duration of working in MOH, mean interaction with researchers, or extent of research use (Additional file).

## Discussion

To our knowledge, this is the first study in the Middle East and North Africa (MENA) region that aimed to explore health policymakers’ capacities, engagement, and use of research in health policymaking. In the MENA region, limited attention has been given to investigating the research capabilities of health policymakers. This paper represents a significant step forward in examining the factors that influence the shaping of health policies in the region. The present study focuses on understanding how Kuwaiti health policymakers incorporate evidence-based research into the formulation of health-related policies.

The analysis of participants' perspectives on the value placed on using research yielded insightful findings related to research utilization within the Kuwait MOH. A mean score of 4.29 ± 0.55 indicated a generally high level of importance attributed to research by the participants. This finding was similar to that of a previous study [4]. Participants showed a universal recognition of the significance of research in their professional context. This collective acknowledgement underscores the foundation of a research-informed approach within the MOH. A key observation is the nuanced perception of the statement "Meet organizational requirements to use research." While labelled neutral by many participants, it was still recognized by the highest number as valuable. This finding suggests a potential misalignment between individual perceptions and organizational expectations, urging further exploration of the underlying factors influencing this discrepancy.

Consistent with the findings of another study [4], the present study participants expressed confidence in their own knowledge and skills for research utilization. However, the finding that "Evaluate the quality of research" received the highest "not confident" and "neutral" labels raised important considerations for capacity-building initiatives. On the positive side, confidence was expressed in applying research to policy or program development and commissioning research, indicating specific areas of strength that can be leveraged for further skill development. These insights are in line with findings by Mirzoev et al., who highlight the necessity of a multifaceted approach to capacity strengthening, involving, training, mentorship, and organizational support mechanisms [1].

The study also shed light on the organizational context of the value the MOH places on research. The identification of specific areas where the organizational emphasis may be lacking, such as collaboration with researchers and the encouragement of new research generation, provides valuable insights for strategic interventions to enhance the research culture within the MOH. Loncarevic et al. also reported that approximately two-thirds of respondents in their study had not been involved in any research activity, either alone or in collaboration with researchers while being part of the policymaking process [4]. This aligns with findings by Makkar et al., who suggest that organizational capacity, including leadership support, dedicated resources, and a culture of learning, significantly impacts the extent to which research is used in policymaking [15].

A notable challenge highlighted by the participants is the perceived limited processes for policy or program development guidance on how research should be used. This finding stresses the importance of addressing infrastructural barriers to research engagement and utilization within the MOH. The low number of participants recognizing well-developed processes and adequate training opportunities underlines the need for organizational investments in these critical areas. Participants' strategies in accessing and utilizing research demonstrate a nuanced decision-making process. A majority actively sought synthesized research, preferring reviews that evaluate multiple studies and showing an appreciation for comprehensive insights. Concurrently, a significant portion engaged in collaborative efforts, leading or delegating teams to evaluate studies collectively. This approach reflects a thoughtful and comprehensive method, combining in-depth study examination with the exploration of authoritative sources.

A significant association was identified between gender and the organization’s perception of research use. Notably, compared with females, organizations place less value on research utilization among males. The lower emphasis on research utilization in males than in females may stem from various factors, such as organizational culture, leadership styles, or gender-specific roles within the organization. These findings warrant further exploration to understand the underlying causes and implications of gender-inclusive strategies promoting research utilization. Additionally, positive moderate correlations emerged between age, duration of working at the MOH, and the organization’s emphasis on research use. This suggests that as individuals grow older or accumulate more experience within the Ministry of Health, there is a tendency for a greater perception that the organization places a greater value on research utilization. This correlation may indicate a maturation process in recognizing the importance of research in decision-making over one’s career.

Engagement in research appraisal activities shows robust competency in critically evaluating research quality. The diversity of research-generation activities displayed adaptability and recognition of the need for tailored research endeavours. The identified gaps in collaborative grant applications and limited participation in research result analysis or publication activities highlight opportunities for fostering a more vibrant research community within the MOH. The mean score and varied collaborative involvement frequencies indicate diverse engagement styles. Nuanced perceptions about research use in decision-making contexts underscore a thoughtful consideration of research impact. The prevalence of conceptual research over organization-imposed research reflects similar findings from a previous study [4]. Conceptual research often provides a theoretical foundation and conceptual framework for understanding complex issues. Health policies often require a clear understanding of concepts and their interrelationships. Therefore, conceptual research helps clarify these concepts, which can be crucial for policymakers in making informed decisions.

Furthermore, strategies to effectively bring research evidence into policy are crucial, as synthesized by Erismann et al. Their study highlights practical strategies such as engaging stakeholders throughout the research process, building strong networks, and ensuring clear communication of research findings [16]. Mazzucca et al. also identified a significant relationship between state health department practitioners' perceptions of organizational support and their evidence-based decision-making skills, reinforcing the need for strong organizational support [3]. These strategies are essential for the MOH in Kuwait to enhance the integration of research into policymaking. By fostering stakeholder engagement and improving communication channels, the MOH can ensure that research findings are effectively translated into policy actions. Overall, these findings emphasize the importance of creating a supportive environment within the MOH to enhance research utilization. By addressing the identified gaps in collaboration, training, and infrastructure, the MOH can foster a culture that values and integrates research into the policy development process. This approach aligns with Williamson et al.'s emphasis on structured capacity-building interventions, which can increase the capacity of policy agencies to use research findings effectively [17]. Enhancing organizational support and investing in capacity-building initiatives are crucial steps towards promoting EIP in Kuwait and the broader MENA region.

## Implications for practice

The study's implications for healthcare practice in Kuwait are multifaceted, offering strategic insights for cultivating an evidence-informed culture within the MOH. Given the participants' high research valuation, initiatives must be undertaken to align individual perceptions with organizational expectations, particularly in meeting research requirements. Targeted capacity-building programs addressing confidence disparities, specifically in appraising research quality, are crucial for enhancing professionals' skills and promoting effective evidence evaluation.

In light of the identified organizational gaps, fostering collaboration with researchers and encouraging new research generation are imperative. Creating platforms for interaction and incentivizing internal research initiatives can nurture a more dynamic research ecosystem within the MOH. Addressing challenges related to limited access to training in using research and systems that encourage leaders to support the use of research necessitates organizational investments in infrastructure and technology, enhancing professionals' ability to access and utilize diverse research sources.

Ultimately, these strategic interventions have the potential to elevate evidence-based decision-making within the Kuwait healthcare context, contributing to improved policy and program development. By clarifying expectations, enhancing skills, fostering collaboration, and addressing infrastructure gaps, the MOH can advance its mission of delivering healthcare services grounded in the latest and most relevant related research.

## Strength and limitations

This study has several strengths. First, this study represents the initial comprehensive evaluation of individual research engagement and utilization capacity in Kuwait and the Middle East at large. Second, this study pioneered the application of the validated SEER measure. This approach aids in identifying areas of vulnerability in the health policy process concerning research utilization. Such insights can inform targeted strategies for capacity building. Third, the survey included health policymakers from diverse organizational levels with varied public health responsibilities, enhancing the generalizability of the findings. The results delineated the aspects of individual capacity that either facilitated or impeded research utilization, indicating areas for improvement.

Nevertheless, the study had its limitations. Survey nonresponse is a perpetual concern, particularly when nonrespondents differ from participants in some discernible or indiscernible manner. The lack of information on response rates and differences between participants and nonparticipants makes it inappropriate to extrapolate from the sample to the broader population. The likelihood that those with greater interest and motivation participated may have led to overestimating the value Kuwaiti policymakers assign to research in policymaking and their perceived ability to integrate research systematically. Although the study targeted all health policymakers, the possibility of the existence of selection bias must be acknowledged.

## Conclusion

In conclusion, this study provides a comprehensive overview of the factors influencing research utilization within the MOH, encompassing individual perspectives, organizational priorities, and engagement with research activities. The identified areas of strength and challenges pave the way for targeted interventions aimed at optimizing the integration of research into policy and program development within the MOH.

### Supplementary Information

Supplementary Material 1.

## Data Availability

The dataset supporting the conclusions of this article is included within the article and its additional files.

## Abbreviations

MOH: Kuwait Ministry of Health
SEER: Seeking, Engaging with and Evaluating Research
