# Supplementary material for: Building bridges: evaluating policymakers' research capacities, engagement, and utilization in health policymaking within the Kuwaiti context: a cross-sectional study
Source: Health Res Policy Syst. 2024 Jul 15;22:84. doi: 10.1186/s12961-024-01177-9 (PMC11247873; doi:10.1186/s12961-024-01177-9)
Supplement: Supplementary file 1 — Supplementary Material 1. [file 12961_2024_1177_MOESM1_ESM.docx]

**Additional file:**

Table: Demographics of the participants

|  |  | N (%) |
| --- | --- | --- |
| Age | **Mean ± SD** | 49.84 ± 7.28 |
| Gender | Male | 45 (51.1%) |
|  | Female | 43 (48.9%) |
| Marital status | Single | 15 (17%) |
|  | Married | 69 (78.4%) |
|  | Divorced | 2 (2.3%) |
|  | Separated | 2 (2.3%) |
| Nationality | Kuwaiti | 87 (98.9%) |
|  | Non-Kuwaiti | 1 (1.1%) |
| Highest qualification | Bachelor’s degree | 12 (13.6%) |
|  | Diploma | 9 (10.2%) |
|  | Master’s degree | 10 (11.4%) |
|  | Board or Fellowship | 29 (33%) |
|  | MD | 7 (8%) |
|  | PhD | 21 (23.9%) |
| Country of highest qualification | Kuwait | 40 (45.5%) |
|  | Western and other countries | 38 (43.2%) |
|  | Arab (Egypt, Saudi Arabia, or Bahrain) | 10 (11.4%) |
| Duration of working in MOH (In years) | **Mean ± SD** | 24.39 ± 6.80 |

Table: Value that individuals place on using research, confidence that individuals have in their own knowledge and skills, and value organization places on using research

|  |  | Not at all valuable | Not valuable | Neutral | Valuable | Very valuable | Mean ± SD |
| --- | --- | --- | --- | --- | --- | --- | --- |
| Value individual places on using research | Identify issues that require a policy or program response | 0 (0%) | 2 (2.3%) | 4 (4.5%) | 42 (47.7%) | 40 (45.5%) | 4.29 ± 0.55 |
|  | Understand how to think about issues | 0 (0%) | 2 (2.3%) | 5 (5.7%) | 43 (48.9%) | 38 (43.2%) |  |
|  | Decide about content or direction of a policy or program | 0 (0%) | 2 (2.3%) | 10 (11.4%) | 41 (46.6%) | 35 (39.8%) |  |
|  | Persuade others to a point of view or course of action | 0 (0%) | 3 (3.4%) | 7 (8%) | 44 (50%) | 34 (38.6%) |  |
|  | Design the implementation or evaluation strategy for a policy or program | 0 (0%) | 1 (1.1%) | 8 (9.1%) | 38 (43.2%) | 41 (46.6%) |  |
|  | Monitor implementation or evaluate the impact of a policy or program | 0 (0%) | 1 (1.1%) | 6 (6.8%) | 38 (43.2%) | 43 (48.9%) |  |
|  | Meet organizational requirements to use research | 1 (1.1%) | 1 (1.1%) | 13 (14.8%) | 48 (54.5%) | 25 (28.4%) |  |
|  |  | **Not at all confident** | **Not confident** | **Neutral** | **Confident** | **Very confident** | **Mean ± SD** |
| Confidence individual has in their own knowledge and skills | Find research to inform policy or program development | 1 (1.1%) | 6 (6.8%) | 16 (18.2%) | 41 (46.6%) | 24 (27.3%) | 3.78 ± 0.70 |
|  | Evaluate the quality of research | 0 (0%) | 11 (12.5%) | 22 (25%) | 37 (42%) | 18 (20.5%) |  |
|  | Interpret the results of research | 0 (0%) | 6 (6.8%) | 21 (23.9%) | 45 (51.1%) | 16 (18.2%) |  |
|  | Apply research to policy or program development | 0 (0%) | 5 (5.7%) | 18 (20.5%) | 48 (54.5%) | 17 (19.3%) |  |
|  | Design evaluations of policies or programs | 1 (1.1%) | 10 (11.4%) | 18 (20.5%) | 47 (53.4%) | 12 (13.6%) |  |
|  | Commission research to support policy or program development | 0 (0%) | 10 (11.4%) | 21 (23.9%) | 48 (54.5%) | 9 (10.2%) |  |
|  | Partner with researchers to generate research | 0 (0%) | 5 (5.7%) | 18 (20.5%) | 48 (54.5%) | 17 (19.3%) |  |
|  |  | **Never** | **Rarely** | **Sometimes** | **Often** | **Always** | **Mean ± SD** |
| Value organization places on using research | Leaders believe it is important to use research in policy or program development | 3 (3.4%) | 12 (13.6%) | 32 (36.4%) | 26 (29.5%) | 15 (17%) | 3.30 ± 0.88 |
|  | It is expected that research will be used in policy or program development | 2 (2.3%) | 17 (19.3%) | 31 (35.2%) | 27 (30.7%) | 11 (12.5%) |  |
|  | Generation of new research to inform policy or program development is encouraged | 4 (4.5%) | 23 (26.1%) | 26 (29.5%) | 26 (29.5%) | 9 (10.2%) |  |
|  | It is expected that policies/programs will be evaluated | 1 (1.1%) | 17 (19.3%) | 32 (36.4%) | 24 (27.3%) | 14 (15.9%) |  |
|  | Interaction or collaboration with researchers or research organizations is encouraged | 7 (8%) | 17 (19.3%) | 27 (30.7%) | 25 (28.4%) | 12 (13.6%) |  |

For each question, a score was given to the answers ranging from 1 to 5, giving a possible score for each domain ranging from 1 to 5, which is calculated as the mean of all questions of the domain.

Table: Tools and systems organization has to support research engagement actions and use

|  |  | No | Yes, but limited | Yes, well developed | I don’t know | Mean ±SD |
| --- | --- | --- | --- | --- | --- | --- |
| Tools and systems organization has to support research engagement actions and use | Has processes for policy or program development that provide guidance on how research should be used | 19 (21.6%) | 50 (56.8%) | 13 (14.8%) | 6 (6.8%) | 1.67± 0.54 |
|  | Has systems that encourage leaders to support use of research | 27 (30.7%) | 44 (50%) | 8 (9.1%) | 9 (10.2%) |  |
|  | Provides access to training in using research in policy or program development | 26 (29.5%) | 41 (46.6%) | 13 (14.8%) | 8 (9.1%) |  |
|  | Has the resources needed to access research (e.g., subscriptions to journals, a library, relevant software) | 47 (53.4%) | 24 (27.3%) | 10 (11.4%) | 7 (8%) |  |
|  | Has established methods for commissioning reviews of research | 37 (42%) | 25 (28.4%) | 10 (11.4%) | 16 (18.2%) |  |
|  | Has documented processes for how policies or programs should be evaluated | 31 (35.2%) | 38 (43.2%) | 11 (12.5%) | 8 (9.1%) |  |
|  | Has existing relationships, or established methods for engaging, with research organizations | 24 (27.3%) | 37 (42%) | 13 (14.8%) | 14 (15.9%) |  |

For each question, a score was given to the answers ranging from 1 to 3, no (1), yes but limited (2), yes well developed (3), I don’t know (1), giving a possible score for each domain ranging from 1 to 3 which is calculated as the mean of all questions of the domain.

Table: Access to synthesized research

|  |  | No | Yes |
| --- | --- | --- | --- |
| Accessed synthesized research | Search for reviews of research summarizing and evaluating the results of multiple studies (i.e., systematic reviews, meta-analyses)? | 26 (29.5%) | 62 (70.5%) |
|  | Headed or delegated a team to summarize and evaluate the results of available studies? | 35 (39.8%) | 53 (60.2%) |

Table: Access to primary research

|  |  | No | Yes |
| --- | --- | --- | --- |
| Accessed primary research | Search for research papers reporting the results of single studies (e.g., randomized controlled trials, qualitative studies)? | 30 (34.1%) | 58 (65.9%) |
|  | Search for research on government websites? | 41 (46.6%) | 47 (53.4%) |

Table: Appraised research

|  |  | No | Yes |
| --- | --- | --- | --- |
| Appraised research | The appropriateness of methods used to answer the question | 32 (36.4%) | 56 (63.6%) |
|  | The likelihood that the methods used meant that the results were reliable (unbiased) | 30 (34.1%) | 58 (65.9%) |
|  | Generalizability of the findings to your context, based on similarity of the included population, health system or other factors | 28 (31.8%) | 60 (68.2%) |

Table: Generated research questionnaire

|  |  | No | Yes |
| --- | --- | --- | --- |
| Generated research | Undertake or participate in an internally conducted research project or analysis of data | 27 (30.7%) | 61 (69.3%) |
|  | Commission or partner with researchers to conduct a research project or analysis of data | 29 (33%) | 59 (67%) |
|  | Plan or undertake an evaluation of the program or policy | 30 (34.1%) | 58 (65.9%) |

Table: Interaction with researchers

|  |  | Not at all | Once | Twice | More than twice | Mean ± SD |
| --- | --- | --- | --- | --- | --- | --- |
| Interacted with researchers | Worked with researchers to identify policy or program direction/priorities, or research direction/priorities | 39 (44.3%) | 19 (21.6%) | 9 (10.2%) | 21 (23.9%) | 2.07 ± 0.92 |
|  | Collaborated with researchers to develop or implement a research project | 33 (37.5%) | 19 (21.6%) | 15 (17%) | 21 (23.9%) |  |
|  | Collaborated on a competitive research grant application (e.g., KFAS) | 61 (69.3%) | 14 (15.9%) | 6 (6.8%) | 7 (8%) |  |
|  | Contributed to analysis and/or writing up of research results, or to other aspects of a research publication | 46 (52.3%) | 21 (23.9%) | 8 (9.1%) | 13 (14.8%) |  |
|  | Attended forums (e.g., conferences, workshops, symposia) to hear about research findings | 24 (27.3%) | 22 (25%) | 11 (12.5%) | 31 (35.2%) |  |
|  | Acted in an advisory capacity to a research team (e.g., on a steering committee) | 42 (47.7%) | 19 (21.6%) | 7 (8%) | 20 (22.7%) |  |

For each question, a score was given to the answers ranging from 1 to 4 based on the frequency, giving a possible score for the domain ranging from 1 to 4, which is calculated as the mean of all questions.

Table: The extent of research use

|  |  | N/A | None | Minimal | Limited | Moderate | High | Extensive | Mean ± SD |
| --- | --- | --- | --- | --- | --- | --- | --- | --- | --- |
| Extent of research use | Agenda setting/scoping | 9 (10.2%) | 8 (9.1%) | 14 (15.9%) | 13 (14.8%) | 29 (33%) | 13 (14.8%) | 2 (2.3%) | 2.54 ± 1.29 |
|  | Policy or program development | 3 (3.4%) | 6 (6.8%) | 16 (18.2%) | 15 (17%) | 27 (30.7%) | 13 (14.8%) | 8 (9.1%) |  |
|  | Policy or program implementation | 3 (3.4%) | 7 (8%) | 13 (14.8%) | 15 (17%) | 27 (30.7%) | 17 (19.3%) | 6 (6.8%) |  |
|  | Policy or program evaluation | 3 (3.4%) | 10 (11.4%) | 11 (12.5%) | 17 (19.3%) | 20 (22.7%) | 22 (25.0%) | 5 (5.7%) |  |

* For each question, a score was given to the answers ranging from 0 to 6 based on the extent of use, giving a possible score for the domain ranging from 0 to 6, which is calculated as the mean of all questions.

Table: Type of research used

|  |  | No | Yes |
| --- | --- | --- | --- |
| Type of research used | Conceptual research use to help you understand how to think about an issue | 32 (36.4%) | 56 (63.6%) |
|  | Instrumental research use to decide about content or direction of a policy or program | 36 (40.9%) | 52 (59.1%) |
|  | Tactical research use to persuade others to a point of view or course of action | 33 (37.5%) | 55 (62.5%) |
|  | Imposed research use because your organization required you to use research | 52 (59.1%) | 36 (40.9%) |

**Table: Comparison of mean value individual places on using research across participants' demographics**

| Mean value individual places on using research | | N | Mean | SD | P value |
| --- | --- | --- | --- | --- | --- |
| Gender | Male | 45 | 4.20 | 0.65 | 0.124 |
|  | Female | 43 | 4.38 | 0.42 |  |
| Marital status | Single | 15 | 4.43 | 0.38 | 0.428 |
|  | Married | 69 | 4.25 | 0.59 |  |
|  | Divorced | 2 | 4.71 | 0.40 |  |
|  | Separated | 2 | 4.07 | 0.30 |  |
| Highest qualification | Bachelor’s degree | 12 | 4.44 | 0.44 | 0.447 |
|  | Board or Fellowship | 29 | 4.22 | 0.62 |  |
|  | Diploma | 9 | 4.02 | 0.83 |  |
|  | Master’s degree | 10 | 4.27 | 0.46 |  |
|  | MD | 7 | 4.27 | 0.64 |  |
|  | PhD | 21 | 4.42 | 0.35 |  |
| Country of highest qualification | Kuwait | 40 | 4.13 | 0.63 | 0.055 |
|  | Western and other countries | 38 | 4.40 | 0.46 |  |
|  | Arab (Egypt, Saudi Arabia, or Bahrain) | 10 | 4.46 | 0.45 |  |
|  | | **Pearson Correlation** | | | **P value** |
| Age (In years) | | 0.151 | | | 0.163 |
| Duration of working in MOH (In years) | | 0.040 | | | 0.710 |

**Table:** **Comparison of mean value organization places on using research across participants' demographics**

| Mean value organization places on using research | | N | Mean | SD | P value |
| --- | --- | --- | --- | --- | --- |
| Gender | Male | 45 | 3.10 | 0.91 | **0.034*** |
|  | Female | 43 | 3.50 | 0.80 |  |
| Marital status | Single | 15 | 3.57 | 0.62 | 0.538 |
|  | Married | 69 | 3.23 | 0.94 |  |
|  | Divorced | 2 | 3.10 | 0.71 |  |
|  | Separated | 2 | 3.60 | 0.00 |  |
| Highest qualification | Bachelor’s degree | 12 | 3.38 | 1.04 | 0.449 |
|  | Board or Fellowship | 29 | 3.24 | 0.63 |  |
|  | Diploma | 9 | 3.11 | 1.28 |  |
|  | Master’s degree | 10 | 3.70 | 0.57 |  |
|  | MD | 7 | 3.29 | 0.60 |  |
|  | PhD | 21 | 3.21 | 1.09 |  |
| Country of highest qualification | Kuwait | 40 | 3.26 | 0.90 | 0.892 |
|  | Western and other countries | 38 | 3.35 | 0.82 |  |
|  | Arab (Egypt, Saudi Arabia or Bahrain) | 10 | 3.26 | 1.07 |  |
|  | | **Pearson Correlation** | | | **P value** |
| Age (In years) | | 0.297 | | | **0.005*** |
| Duration of working in MOH (In years) | | 0.353 | | | **0.001*** |

*: significant as P value ≤ 0.05

**Table:** **Comparison of individuals’ mean confidence in their own knowledge and skills across participants' demographics**

| Mean confidence individual has in their own knowledge and skills | | N | Mean | SD | P value |
| --- | --- | --- | --- | --- | --- |
| Gender | Male | 45 | 3.69 | 0.64 | 0.196 |
|  | Female | 43 | 3.88 | 0.76 |  |
| Marital status | Single | 15 | 4.05 | 0.48 | 0.179 |
|  | Married | 69 | 3.70 | 0.74 |  |
|  | Divorced | 2 | 4.43 | 0.00 |  |
|  | Separated | 2 | 4.00 | 0.81 |  |
| Highest qualification | Bachelor’s degree | 12 | 3.87 | 0.65 | 0.219 |
|  | Board or Fellowship | 29 | 3.61 | 0.71 |  |
|  | Diploma | 9 | 3.60 | 0.93 |  |
|  | Master’s degree | 10 | 3.74 | 0.39 |  |
|  | MD | 7 | 3.71 | 0.83 |  |
|  | PhD | 21 | 4.10 | 0.65 |  |
| Country of highest qualification | Kuwait | 40 | 3.60 | 0.80 | 0.104 |
|  | Western and other countries | 38 | 3.94 | 0.52 |  |
|  | Arab (Egypt, Saudi Arabia, or Bahrain) | 10 | 3.93 | 0.80 |  |
|  | | **Pearson Correlation** | | | **P value** |
| Age (In years) | | 0.096 | | | 0.376 |
| Duration of working in MOH (In years) | | 0.092 | | | 0.394 |

**Table:** **Comparison of the means of interactions with researcher factors across participant demographics**

| Mean interacted with researchers | | N | Mean | SD | P value |
| --- | --- | --- | --- | --- | --- |
| Gender | Male | 45 | 2.09 | 0.99 | 0.875 |
|  | Female | 43 | 2.05 | 0.85 |  |
| Marital status | Single | 15 | 2.23 | 0.92 | 0.614 |
|  | Married | 69 | 2.04 | 0.93 |  |
|  | Divorced | 2 | 2.42 | 0.12 |  |
|  | Separated | 2 | 1.42 | 0.59 |  |
| Highest qualification | Bachelor’s degree | 12 | 1.89 | 0.88 | 0.285 |
|  | Board or Fellowship | 29 | 2.16 | 0.98 |  |
|  | Diploma | 9 | 1.87 | 0.91 |  |
|  | Master’s degree | 10 | 1.70 | 0.44 |  |
|  | MD | 7 | 2.17 | 0.81 |  |
|  | PhD | 21 | 2.29 | 1.05 |  |
| Country of highest qualification | Kuwait | 40 | 1.99 | 0.96 | 0.278 |
|  | Western and other countries | 38 | 2.23 | 0.93 |  |
|  | Arab (Egypt, Saudi Arabia, or Bahrain) | 10 | 1.77 | 0.56 |  |
|  | | **Pearson Correlation** | | | **P value** |
| Age (In years) | | 0.036 | | | 0.738 |
| Duration of working in MOH (In years) | | 0.050 | | | 0.644 |

**Table:** **Comparison of the mean extent of research use across participants' demographics**

| Mean extent of research use | | N | Mean | SD | P value |
| --- | --- | --- | --- | --- | --- |
| Gender | Male | 45 | 3.23 | 1.43 | 0.398 |
|  | Female | 43 | 3.49 | 1.38 |  |
| Marital status | Single | 15 | 3.57 | 1.34 | 0.498 |
|  | Married | 69 | 3.26 | 1.43 |  |
|  | Divorced | 2 | 4.63 | 1.59 |  |
|  | Separated | 2 | 3.75 | 0.35 |  |
| Highest qualification | Bachelor’s degree | 12 | 3.27 | 1.20 | 0.058 |
|  | Board or Fellowship | 29 | 2.84 | 1.55 |  |
|  | Diploma | 9 | 2.89 | 1.49 |  |
|  | Master’s degree | 10 | 4.03 | 0.94 |  |
|  | MD | 7 | 3.79 | 0.88 |  |
|  | PhD | 21 | 3.86 | 1.38 |  |
| Country of highest qualification | Kuwait | 40 | 3.21 | 1.45 | 0.607 |
|  | Western and other countries | 38 | 3.53 | 1.34 |  |
|  | Arab (Egypt, Saudi Arabia, or Bahrain) | 10 | 3.33 | 1.56 |  |
|  | | **Pearson Correlation** | | | **P value** |
| Age (In years) | | 0.053 | | | 0.628 |
| Duration of working in MOH (In years) | | 0.029 | | | 0.790 |
